# Association of apolipoprotein E polymorphism with plasma lipid disorders, independent of obesity-related traits in Vietnamese children

**DOI:** 10.1186/s12944-016-0349-6

**Published:** 2016-10-10

**Authors:** Nguyen Thi Hong Hanh, Bui Thi Nhung, Duong Thi Anh Dao, Le Thi Tuyet, Le Thi Hop, Tran Quang Binh, Vu Thi Minh Thuc

**Affiliations:** 1 Hanoi National University of Education, 136 Xuan Thuy Street, Hanoi, Vietnam; 2 National Institute of Nutrition, 48B Tang Bat Ho Street, Hanoi, Vietnam; 3 National Institute of Hygiene and Epidemiology, 1 Yersin, Hanoi, Vietnam; 4 National Hospital of Otorhinolaryngology, 78 Giai Phong Road, Hanoi, Vietnam

**Keywords:** *APOE* gene, Association, lipid profiles, Dyslipidemia, Vietnamese children

## Abstract

**Background:**

The dyslipidemia associated with obesity plays a major role in the development of atherosclerosis and cardiovascular disease. Dyslipidemia in childhood can progress in adult stage. *APOE* is one of the most important genes that regulate plasma lipid transport and clearance. The study aimed to assess whether the common *APOE* polymorphism is associated with lipid profiles and dyslipidemia, and it could be modulated by obesity-related traits (body mass index, waist circumference, hip circumference, and waist-to-hip ratio) in Vietnamese children.

**Methods:**

A case-control study was designed including 249 cases with dyslipidemia and 600 controls without dyslipidemia. Dyslipidemia is defined as elevated total or low-density lipoprotein (LDL) cholesterol levels, or low levels of high-density lipoprotein (HDL) cholesterol. Genotype for *APOE* polymorphism (rs7412 and rs429358) was determined by the polymerase chain reaction and restriction fragment length polymorphism method. The association of *APOE* genotypes with plasma lipid disorders was tested by binary logistic regression analysis, taking into account the confounding factors of age, sex, residence, province and obesity-related traits.

**Results:**

In comparison with ε3/ε3 carriers, the ε4 carriers had the highest concentration of serum TC and LDL-C in cases and controls (*P* ≤ 0.001), while ε2 carriers had the lowest. Carriers without TT haplotype had higher serum TC than those with TT haplotype. The ε4 carriers had higher hypoalphalipoproteinemia risk than ε3/ε3 carriers (OR = 2.78, *P* = 0.02) before and after adjustment for age, gender, residence and obesity-related traits.

**Conclusions:**

The study suggested that the *APOE* genotype and haplotype significantly associated with plasma TC and LDL-C level in Vietnamese children. The association of *APOE* genotype with hypoalphalipoproteinemia was independent of obesity-related traits.

**Electronic supplementary material:**

The online version of this article (doi:10.1186/s12944-016-0349-6) contains supplementary material, which is available to authorized users.

## Background

Dyslipidemia, abnormal of lipid levels in the blood, is a leading risk factor for coronary artery disease (CAD), hypertension, and stroke-the main cause of mortality globally [1, 2]. Dyslipidemia includes hypercholesterolemia - high total cholesterol (TC) level, hyperbetalipoproteinemia - high low density lipoprotein-cholesterol (LDL-C) level, hypoalphalipoproteinemia-reduced high density lipoprotein-cholesterol (HDL-C) level and hypertriglyceridemia-elevated triglyceride (TG) level. It is especially alarming that the proportion of children with dyslipidemia is increasing dramatically, proportional to the percentage of overweight-obesity children in both developed and developing countries. Dyslipidemia was observed in 85.3 % of the overweight children and adolescents [3]. Percentages of 4–9 years old Vietnamese overweight-obesity children had hypertriglycemia, hypercholesterolemia, hyperbetalipoproteinemia and hypoalphalipoproteinemia were 30.7, 15.3, 12.6 and 5.3 %, respectively [4]. Dyslipidemia in childhood can progress in adult stage [5]. Therefore, the early detection of lipoprotein disorders at a young age is very important to prevent complications and decrease coronary heart disease risk in future.

Dyslipidemia is well-known as a complex disorder which is influenced by genetics and environmental factors [6–8]. There are many environmental factors related to the plasma lipid profile, such as socioeconomic status, diet and physical activity, and especially obesity. Obesity leads to lipid abnormalities partly mediated by adipokines and free fatty acids [9]. In obese individual, reductions in mRNA expression levels of lipoprotein lipase in adipose tissue and lipoprotein lipase activity in skeletal muscle along with competition for lipolysis between very low density lipoprotein and chylomicrons result in decreased lipolysis of TG-rich lipoproteins [10, 11]. Compared with normal-weight individuals, obese patients present with elevated cholesterol synthesis [12]. Therefore, obesity is probably the main cause of dyslipidemia. The distribution of central fat determined by waist circumference was associated with abnormal of TG, LDL-C, and HDL-C levels in children aged 5–17 years [13]. Besides, genetic factors are considered to be important determinants of plasma concentrations of TC, TG, LDL-C and HDL-C in adults [14]. *APOE* is one of the most important genes that regulate plasma lipid transport and clearance. *APOE* gene encodes for apolipoprotein E (apo E) protein [15]. Apo E is also one of the central and well established regulators of plasma lipid levels by affecting the hepatic binding, uptake, and catabolism of several classes of lipoproteins [16]. The 3 major isoforms of human apo E (E2, E3, and E4) coded by 3 alleles (ε2, ε3, and ε4) differ in amino acid sequence at 2 sites, residue 112 (rs429358) and residue 158 (rs7412) [17]. These differences alter *APOE* structure and function. The ε3 (112 cysteine-Cys, 158 arginine-Arg) allele is considered the “neutral” *APOE* genotype while the ε4 (112 Arg and 158 Arg) and ε2 (112 Cys, 158 Cys) allele associated with dyslipidemia in different adult populations [15, 18–20]. However, whether there is a difference between adults and children in the role of *APOE* for determining plasma lipid and lipoproteins is not clearly understood. Several studies have reported the significant association of *APOE* polymorphisms with lipid profile in Caucasian children [21–24], while studies in Asian children showed inconsistent associations [25–27]. Furthermore, the effect of the *APOE* polymorphism on the lipid profiles has been reported to be modulated by obesity in children [28, 29].

Therefore, we focused on two key questions: (i) Are *APOE* rs429358 and rs7412 polymorphisms significantly associated with lipid profiles and dyslipidemia in Vietnamese children? and (ii) whether the association could be modulated by obesity-related traits?

## Methods

### Study subjects

To identify the association between the common *APOE* polymorphism and plasma lipid disorders in Vietnamese children, we designed a case-control study in which 249 cases were children with hyperlipoproteinemia and 600 controls were children without hyperlipoproteinemia. The study included 849 unrelated children aged 6–10 years: 567 children (405 boys and 162 girls) in Hanoi City; 162 children (104 boys and 58 girls) in Thai Nguyen province and 120 children (81 boys and 39 girls) in Hai Duong province. All of them were Kinh-the major ethnic group in Vietnam. The subjects had no evidence of diseases related to atherosclerosis, coronary artery diseases, diabetes and mental disorder. None of them were using lipid-lowering medication when the blood sample was taken.

### Data collection

A questionnaire was provided along with consent forms and parents were asked to provide information regarding the child’s general health. The body weight of participants was measured to the nearest 0.1 kg with standardized medical scales. Subjects were weighed without shoes and minimum of clothing. The height was measured to the nearest 0.1 cm. Body mass index (BMI) was calculated as weight divided by height squared (kg/m^2^). Waist circumference (WC) was measured at the end of a normal expiration to the nearest 0.1 cm at the mid-point between the last floating rib and the top of the iliac crest. Hip circumference (HC) was measured at the level of the symphysispubis and the greatest gluteal protuberance. Waist-to-hip ratio (WHR) was calculated as WC in cm divided by HC in cm. Blood pressure was measured by using a standardized automated sphygmomanometer (Omron HEM-6131, Tokyo, Japan) after 5 min of rest in the seated position. Blood pressure was measured in duplicate, with an interval of at least 1 min. The mean of the 2 measurements was used as the blood pressure value.

### Lipid analysis

Blood lipids and lipoproteins were measured on samples obtained after an overnight fast. Blood samples were collected in EDTA containing tubes. Serum total cholesterol (TC) and triglyceride (TG) were determined with enzymatic method. Serum high-density lipoprotein-cholesterol (HDL-C) and low-density lipoprotein-cholesterol (LDL-C) were measured through a direct assay. All determinations were performed with autoanalyzer (Type Architect C8000; Abbott Ltd., United States of America).

Dyslipidemia was diagnosed according to the criteria of National Cholesterol Education Program [30] for TC and LDL-C and US National Institutes of Health Heart, Lung, and Blood Institute [31] for HDL-C and TG. Hypertriglyceridemia was defined as TG ≥ 100 mg/dL (with children age under 10 years) or TG ≥ 130 mg/dL (with children age uper 10 years). Hypercholesterolemia was defined as TC ≥ 200 mg/dL. Hyperbetalipoproteinemia was defined as LDL-C ≥ 130 mg/dL. Hypoalphalipoproteinemia was defined as HDL-C ≤ 35 mg/dL. The individuals with at least one of the four criteria above were defined as dyslipidemia.

### Genotyping method

Peripheral blood samples were obtained from each participants and genomic DNA was extracted from peripheral blood leukocytes, using Wizard® Genomic DNA Purification Kit (Promega Corporation, USA). Genotyping of the *APOE* was performed by polymerase chain reaction and restriction fragment length polymorphism (PCR-RFLP) method according to Hixson and Vernier [32] method with some modification. The *Fastdigest Hha*I restriction enzyme (Thermo Corporation, USA) was added directly to the reaction system consisting of 244 base-pair PCR products (3 μL) in a total 8.0 μL digested for 15 min at 37 °C. Each reaction mixture was loaded onto 12 % polyacrylamide gel and electrophoresised for 90 min at 150 V, 20 mA and 10 W. The digested product was visualized by UV illumination after Redsafe staining for 30 min. Each genotype possessed unique combination of *Hha*I fragment size. To confirm the accuracy of the RFLP method, six random samples with different genotypes detected by the RFLP method were also confirmed by direct sequencing. The PCR products were purified by low melting point gel electrophoresis and phenol extraction, and then the DNA sequences were analyzed by using the BigDye® Terminator v3.1 cycle sequencing kit chemistry in Axil Scientific Pte Ltd., Singapore. The genotypes by RFLP method were in completed concordance with those of the sequencing method.

### Statistical analysis

Qualitative variables were expressed as percentages. Quantitative variables were expressed as means ± SD if variables were normal distribution or median (interquartile range) if variables were not normal distribution. Chi-square test or one-way ANOVA or independent-sample *t* test or Kruskal-Wallis test or Mann-Whitney *U* test were used when appropriate. Allele frequency was determined for Hardy-Weinberg equilibrium by Fisher’s exact test. The association of genotypes with plasma lipid disorders was tested by binary logistic regression analysis, taking into account the confounding factors of age, sex, residence, province and each of obesity-related traits including body mass index (BMI), waist circumference (WC), hip circumference (HC), and waist-to-hip ratio (WHR). The statistical analyses were done with the statistical software package SPSS16.0 (SPSS Inc., Chicago, Illinois). To adjust the threshold below which a *P*-value is considered significant for multiple testing, we used a Bonferroni correction separately for each group of similar tests, taken into account the linkage disequilibrium between two SNPs and correlation among phenotypes. Specifically, *P*-values < 0.0125 were considered as significant when comparing serum lipid parameters among *APOE* genotypes and haplotypes; *P*-values < 0.025 were considered as significant when evaluating risk factors for plasma lipid disorders.

## Results

Lipid profile and anthropometric characteristics of the subjects in case and control groups are presented in Table 1. There were significant differences between dyslipidemia and control groups in weight, BMI, WC, WHR, TG, TC, LDL-C, and HDL-C. The genotypic distributions for rs429358 and rs7412 were in Hardy-Weinberg equilibrium in controls (*P >* 0.05). There was the strong linkage disequilibrium between two SNPs (D’ = 0.99, *P* = 0.0001). TC haplotype had the highest frequency in all groups (cases: 83.8 %; controls: 80.5 %). Frequencies of TT and CC haplotypes were, respectively, 9.8 and 9.6 % in cases, and 9.0 and 7.2 % in controls.

**Table 1:** Characteristics of the study subjects

Parameter	Controls (*N* = 600)	Cases (*N* = 249)	*P-*value
Male (%)	70.9	67.3	0.304
Age (years)^b^	7.9 (6.9–9.0)	7.9 (6.9–8.7)	0.811
Weight (kg)^b^	28.4 (22.1–36.0)	30.9 (23.6–38.1)	**0.010**
Height (cm)^a^	128.8 ± 9.2	126.6 ± 9.1	0.744
BMI (kg/m^2^)^b^	16.8 (15.0–21.8)	19.6 (15.6–22.9)	**<0.0001**
Waist circumference (cm)^b^	56.0 (51.5–67.5)	67.2 (55.7–73.9)	**<0.0001**
WHR^a^	0.87 ± 0.065	0.89 ± 0.068	**<0.0001**
Systolic blood pressure (mmHg)^b^	107.0 (100.0–115.0)	109.0 (100.0–117.0)	0.210
Diastolic blood pressure (mmHg)^b^	68.0 (60.0–74.0)	69.0 (60.0–75.5)	0.608
Triglyceride (mg/dL)^b^	62.0 (48.7–77.9)	120.4 (103.5–149.6)	**<0.0001**
Total cholesterol (mg/dL)^b^	147.1 (131.6–163.5)	160.8 (141.5–188.3)	**<0.0001**
HDL-C (mg/dL)^b^	54.4 (47.1–62.2)	47.1 (38.9–56.8)	**<0.0001**
LDL-C (mg/dL)^b^	82.6 (72.3–96.3)	94.4 (78.1–119.1)	**<0.0001**

*BMI* body mass index, *WHR* waist to hip ratio, *HDL-C* high-density lipoprotein-cholesterol, *LDL-C* low-density lipoprotein-cholesterol, *TC* total cholesterol

*P*-values obtained by Student *T* test or Mann–Whitney *U* test or Chi-square test. Bold values indicate significant difference between cases and controls

^a^Data are mean ± SD

^b^Data are median (interquartile range)

Lipid profile in cases and controls according to *APOE* genotype is shown in Table 2. The ε4 carriers had the highest concentration of serum TC and LDL-C in cases and controls (*P* ≤ 0.001), while the ε2 carriers had the lowest. The statistically significant differences among the six genotype groups (ε2/ε2, ε2/ε3, ε3/ε3, ε2/ε4, ε3/ε4, and ε4/ε4) were found in TC and LDL-C for both controls and cases (*P* ≤ 0.001). Lipid profile in cases and controls according to *APOE* haplotype is presented in Additional file 1. In both cases and controls, there was a significant difference of TC and LDL-C levels according copy number of TT haplotype: Carriers without TT haplotype had higher serum TC than those with TT haplotype; carriers without TT haplotype had the highest LDL-C level in cases (*P* < 0.0001).

**Table 2:** Lipid profiles in cases and controls according to *APOE* genotypes in Vietnamese children (mg/dL)

	ε2 carriers	ε3/ε3 carriers	ε4 carriers	*P*-value
Controls (*N* = 600)				
TG	62.0 (49.3–79.7)	62.0 (48.7–75.2)	64.6 (51.3–79.7)	0.319
TC	137.1 (123.0–151.9)	148.3 (134.6–165.4)	149.6 (135.8–169.2)	**<0.0001**
HDL-C	54.4 (47.3–64.1)	54.8 (47.2–62.2)	52.1 (45.9–59.1)	0.236
LDL-C	74.1 (69.9–89.3)	83.9 (72.8–96.6)	91.3 (76.6–104.4)	**<0.0001**
Cases (*N* = 249)				
TG	131.0 (106.2–156.6)	117.3 (102.7–142.9)	122.1 (84.7–150.5)	0.520
TC	146.5 (126.9–167.9)	161.5 (141.4–186.5)	177.7 (153.6–195.9)	**0.001**
HDL-C	47.1 (38.9–60.7)	47.8 (39.6–56.4)	44.7 (35.8–54.7)	0.268
LDL-C	77.3 (70.1–99.4)	96.9 (81.2–117.8)	112.1 (86.0–133.6)	**<0.0001**

*TG* triglyceride, *TC* total cholesterol, *HDL-C* high-density lipoprotein-cholesterol, *LDL-C* low-density lipoprotein-cholesterol

Data are median (interquatile range). *P*-values obtained by Kruskall-Wallis test

Bold values indicate a statistically significant difference among *APOE* genotypes after adjustment for multiple testing (*P*-values < 0.0125)

We considered the relationship of some obesity-related traits on blood lipid metabolism disorders, and the results showed that there was a strong association of BMI, WC, HC, WHR with TG, TC, HDL-C, and LDL-C (*P* < 0.0001) (data not shown). Therefore, the association between *APOE* polymorphism and dyslipidemia was further adjusted for obesity-related traits (BMI, WC, HC, and WHR). Table 3 shows that *APOE* genotype was significantly associated with hypoalphalipoproteinemia before and after adjusting for BMI and other covariates (age, gender, residence, and province). The ε4 carriers had higher hypoalphalipoproteinemia risk than theε3/ε3 carriers (OR = 2.78, *P* = 0.02). These above similar associations were found after adjusting for other obesity-related traits including WC, HC, and WHR (Additional file 2).

**Table 3:** Univariate and multivariate analysis of association of *APOE* genotype with dyslipidemia and its components*P*-values obtained by univariate logistic regression

		OR (95 % CI)	*P-*value	OR^*^ (95 % CI)	*P* ^*^-value
*Hypoalphalipoproteinemia*					
*APOE genotype*	ε3/ε3	1		1	
	ε2 carrier	1.19 (0.43–3.26)	0.739	1.29 (0.45–3.63)	0.635
	ε4 carrier	2.68 (1.17–6.14)	**0.020**	2.78 (1.18–6.58)	**0.020**
*Hyperbetalipoproteinemia*					
*APOE genotype*	ε3/ε3	1		1	
	ε2 carrier	0.86 (0.29–2.56)	0.785	1.06 (0.33–3.35)	0.923
	ε4 carrier	2.69 (1.22–5.94)	**0.014**	2.08 (0.85–5.10)	0.110
*Hypertriglyceridemia*					
*APOE genotype*	ε3/ε3	1		1	
	ε2 carrier	1.12 (0.72–1.74)	0.617	1.16 (0.72–1.86)	0.538
	ε4 carrier	1.15 (0.71–1.86)	0.575	1.04 (0.62–1.75)	0.869
*Hypercholesterolemia*					
*APOE genotype*	ε3/ε3	1		1	
	ε2 carrier	0.64 (0.22–1.88)	0.421	0.76 (0.26–2.26)	0.624
	ε4 carrier	1.82 (0.83–4.00)	0.138	1.62 (0.70–3.77)	0.259
*rs429358 Dominant*	T/T	1		1	
Dyslipidemia					
*APOE genotype*	ε3/ε3	1		1	
	ε2 carrier	1.06(0.70–1.60)	0.784	1.14 (0.73–1.77)	0.564
	ε4 carrier	1.36 (0.89–2.08)	0.153	1.26 (0.80–1.99)	0.312

P*-values obtained by multivariate logistic regression and adjusted for age, gender, BMI, residence and province

Bold values indicate a statistically significant after adjustment for multiple testing (*P*-values < 0.025)

## Discussion

We have examined the possible association of the *APOE* rs429358 and rs7412 polymorphism with lipid profiles and disorders in 600 normal and 249 dyslipidemia children. The result showed that the *APOE* genotype was an independent risk factor for hypoalphalipoproteinemia.

Obesity is the main cause of dyslipidemia because obesity leads to impaired peripheral trapping and increased fluxes of free fatty acids from adipocytes to the liver and other tissues as well as hepatic overproduction of very low density lipoprotein, decreased circulating TG lipolysis and the formation of small dense LDL [9]. The effect of the *APOE* polymorphism on the TC/HDL-C and apo A-I/apo B ratios is modulated by BMI z-score and adiponectin levels [29]. Therefore, it is crucial to investigate the association of *APOE* with dislylidemia, considering the contribution of obese status. In present study, the significant association of *APOE* genotype with hypoalphalipoproteinemia has been found before and after adjustment of obesity-related traits in Vietnamese primary school children. The ε4 carriers had higher hypoalphalipoproteinemia risk than the ε3/ε3 carriers.

Regarding the relationship of *APOE* polymorphism with plasma lipid levels, we have evaluated the influence of genotype and haplotype. In both cases and controls, carriers without TT haplotype had higher serum TC than those with TT haplotype. Carriers without TT haplotype also had the highest serum LDL-C in cases. The *APOE* genotype analysis in our study indicated that the ε4 carriers had the highest serum TC and LDL-C concentrations. There are several studies supporting the association of this *APOE* genoype with these plasma lipid concentrations in children. For instance, ε4 carriers had higher TC, TG, LDL-C concentrations, and lower HDL-C concentration, compared with ε3 carriers [23, 24, 29]. The present study observed no statistically significant association between ε4 genotype and HDL-C concentrations, in line with some studies [33, 34]. On the other hand, there were some investigations reporting that HDL-C concentration was statistically lower in ε4 carriers and higher in ε2 carriers [35, 36]. In this study, we also reported that the ε2 carriers had the lowest serum TC and LDL-C. The ε2 allele was associated with lower TC and LDL-C and with higher HDL-C in the 21-year follow-up study from childhood to adulthood in Young Finns Study [23]. There has been a variation in the association between ε2 genotype and TG level among populations. Several studies showed the significant association between ε2 genotype and TG level in Caucasian subjects [37, 38]. On the other hand, in line with our report, several studies indicated that there was no association between ε2 genotype and TG concentration in both Caucasian and Asian subjects [24, 26]. Thus, the association of ε2 genotype with TG concentration varies by ethnicity. Taken together, the variant determination of *APOE* genotype on plasma lipid profile may be due to demographic history, the age of the study subjects or diet.

The isoforms of apoE display different role in lipid metabolism and preferences for specific classes of lipoproteins [39]. The apoE isoforms differ in their ability to interact with some receptors (LDL receptor, the LDL receptor-related protein, the VLDL receptor, the apoE receptor-2, and gp330). The E4 isoform has a higher affinity than E2 and E3 isoforms [40]. The different isoforms vary in cholesterol absorption and postprandial remnant clearance. As a result, this leads to down-regulation of hepatic LDL-receptors in subjects with E4-isoform and an increase in serum cholesterol levels [41, 42]. In addition, ApoE isoform-specific activities may be explained by the intramolecular domain interaction of the N-terminal (receptor binding region) and C-terminal (lipid-binding region) domains to determine the preference of apoE4 for VLDL and of apoE3 and apoE2 for HDL [39].

To the best of our knowledge, our study is the initial report on the potential association of *APOE* genotype and haplotype with plasma lipid disorders in Vietnamese children, independent of obesity-related traits. By comparison with adults, children are less influenced by confounding environmental factors such as alcohol consumption, use of medication, and the presence of undetected diseases. They may therefore be better subjects in whom to identify genetic factors involved in phenotypic variability. The strength of the study is that the sample size was fairly large and it could have the powers between 0.85 and 0.92 to detect the association reported. Meanwhile, there is a limitation of this study. We have not yet considered the influence of diet and physical activity to the association of the *APOE* polymorphism with dyslipidemia in Vietnamese children.

## Conclusions

In conclusion, our data reported that the *APOE* genotype and haplotype were significantly associated with plasma TC and LDL-C level in Vietnamese children; the association of *APOE* genotype with hypoalphalipoproteinemia was independent of obesity-related traits.

## Additional files

Additional file 1:
**Table S1.** Lipid profiles in cases and controls according to *APOE* haplotypes in Vietnamese children (mg/dL). TG, triglyceride; TC, total cholesterol; HDL-C, high-density lipoprotein-cholesterol; LDL-C, low-density lipoprotein-cholesterol. Data are median (interquatile range). *P*-values obtained by Kruskall-Wallis test. Bold values indicate a statistically significant difference among copy number of TC, TT, and CC haplotype after adjustment for multiple testing (*P*-values < 0.0125). (DOCX 19 kb)
Additional file 2:
**Table S2.** Multivariate analysis of association for dyslipidemia, adjusted for age, gender, residence, province and obesity-related traits. *P*-values obtained by multivariate logistic regression and adjusted for age, gender, residence, province, and waist circumference (*P**-values) or hip circumference (*P***-values) or waist-to-hip ratio (*P****-values). Bold values indicate a statistically significant after adjustment for multiple testing (*P*-values < 0.025). (DOCX 19 kb)

## Abbreviations

Apo: Apolipoprotein
BMI: Body mass index
CAD: Coronary artery disease
CI: Confidence interval
DNA: Deoxyribonucleic acid
HC: Hip circumference
HDL-C: High-density lipoprotein-cholesterol
LDL-C: Low-density lipoprotein-cholesterol
OR: Odd ratio
PCR: Polymerase chain reaction
RFLP: Restriction fragment length polymorphism
SNP: Single nucleotide polymorphism
TC: Total cholesterol
TG: Triglyceride
WC: Waist circumference
WHR: Waist to hip ratio
