# Supplementary material for: Association of apolipoprotein E polymorphism with plasma lipid disorders, independent of obesity-related traits in Vietnamese children
Source: Lipids Health Dis. 2016 Oct 10;15:176. doi: 10.1186/s12944-016-0349-6 (PMC5057250; doi:10.1186/s12944-016-0349-6)
Supplement: Additional file 1: — Table S1. Lipid profiles in cases and controls according to APOE haplotypes in Vietnamese children (mg/dL). TG, triglyceride; TC, total cholesterol; HDL-C, high-density lipoprotein-cholesterol; LDL-C, low-density lipoprotein-cholesterol. Data are median (interquatile range). P-values obtained by Kruskall-Wallis test. Bold values indicate a statistically significant difference among copy number of TC, TT, and CC haplotype after adjustment for multiple testing (P-values < 0.0125). (DOCX 19 kb) [file 12944_2016_349_MOESM1_ESM.docx]

**Table S1.** Lipid profiles in cases and controls according to *APOE* haplotypes in Vietnamese children (mg/dL)

| Lipid profile | | Controls (N = 600) | | | | Cases (N =249) | | | |
| --- | --- | --- | --- | --- | --- | --- | --- | --- | --- |
| TC haplotype | | |  |  |  |  |  |  |  |
|  | 0 copy  (N=18) | | 1 copy  (N=158) | 2 copy  (N=142) | *P*-value | 0 copy  (N=14) | 1 copy  (N=69) | 2 copy  (N=166) | *P*-value |
| TG | 71.2 (46.9-79.2) | | 62.8 (49.6-79.7) | 62.0 (48.7-75.2) | 0.399 | 142.9 (121.0-171.5) | 122.1 (96.0-155.3) | 117.3 (102.7-142.9) | 0.028 |
| TC | 147.1 (134.6-165.8) | | 143.7 (128.7-156.8) | 148.3 (134.6-165.4) | 0.051 | 146.3 (118.7-154.8) | 163.8 (142.7-192.5) | 161.5 (141.4-186.5) | 0.036 |
| HDL-C | 55.4 (50.2-73.1) | | 53.5 (46.7-61.2) | 54.8 (47.2-62.2) | 0.264 | 38.9 (36.8-47.0) | 47.1 (37.7-58.3) | 47.8 (39.6-56.4) | 0.215 |
| LDL-C | 89.3 (72.9-97.7) | | 78.5 (71.2-96.0) | 83.9 (72.8-96.6) | 0.206 | 75.2 (72.5-100.3) | 99.0 (75.4-124.7) | 96.9 (81.2-117.8) | 0.203 |
| TT haplotype | | |  |  |  |  |  |  |  |
|  | 0 copy  (N=499) | | 1 copy  (N=94) | 2 copy  (N=7) | P-value | 0 copy  (N=206) | 1 copy  (N=37) | 2 copy  (N=6) | P-value |
| TG | 62.0 (48.7-76.1) | | 62.0 (48.7-79.7) | 60.2 (47.8-78.8) | 0.835 | 118.6 (101.8-145.1) | 134.5 (105.8-160.2) | 131.9 (117.0-146.5) | 0.273 |
| TC | 148.5 (134.6-165.4) | | 138.5 (124.2-152.1) | 142.3 (112.7-169.2) | **<0.0001** | 163.8 (143.0-190.8) | 151.5 (128.5-169.4) | 134.6 (105.1-148.4) | **0.001** |
| HDL-C | 54.4 (47.1-62.2) | | 54.4 (48.0-64.1) | 60.3 (46.7-75.1) | 0.640 | 47.1 (38.9-55.2) | 47.1 (39.7-58.7) | 40.8 (38.5-64.6) | 0.721 |
| LDL-C | 84.7 (73.1-97.1) | | 74.2 (70.3-89.3) | 90.1 (71.2-96.3) | **<0.0001** | 100.4 (81.6-120.4) | 79.3 (71.3-103.1) | 73.3 (52.2-77.3) | **<0.0001** |
| CC haplotype | | |  |  |  |  |  |  |  |
|  | 0 copy  (N=518) | | 1 copy  (N=78) | 2 copy  (N=4) | P-value | 0 copy  (N=205) | 1 copy  (N=40) | 2 copy  (N=4) | P-value |
| TG | 62.0 (48.7-76.1) | | 63.7 (49.6-79.7) | 77.4 (72.3-86.5) | 0.120 | 118.6 (103.5-144.7) | 121.7 (84.7-165.1) | 146.9 (127.0-167.5) | 0.305 |
| TC | 146.5 (130.8-162.0) | | 150.0 (136.3-169.4) | 141.5 (134.6-158.8) | 0.191 | 157.7 (141.0-182.1) | 177.7 (152.7-199.4) | 142.1 (112.4-183.7) | 0.013 |
| HDL-C | 54.4 (47.3-62.3) | | 54.1 (46.3-61.6) | 49.8 (44.6-53.2) | 0.322 | 47.8 (39.5-57.6) | 45.3 (35.4-54.7) | 37.7 (35.9-43.7) | 0.099 |
| LDL-C | 81.4 (71.9-95.9) | | 92.2 (76.6-104.5) | 80.8 (70.8-92.6) | **0.003** | 91.3 (77.3-115.2) | 109.4 (84.1-133.6) | 107.7 (77.7-138.3) | 0.034 |

TG, triglyceride; TC, total cholesterol; HDL-C, high-density lipoprotein-cholesterol; LDL-C, low-density lipoprotein-cholesterol. Data are median (interquatile range). *P*-values obtained by Kruskall-Wallis test. Bold values indicate a statistically significant difference among copy number of TC, TT, and CC haplotype after adjustment for multiple testing (*P*-values < 0.0125).
