# Supplementary material for: Association of apolipoprotein E polymorphism with plasma lipid disorders, independent of obesity-related traits in Vietnamese children
Source: Lipids Health Dis. 2016 Oct 10;15:176. doi: 10.1186/s12944-016-0349-6 (PMC5057250; doi:10.1186/s12944-016-0349-6)
Supplement: Additional file 2: — Table S2. Multivariate analysis of association for dyslipidemia, adjusted for age, gender, residence, province and obesity-related traits. P-values obtained by multivariate logistic regression and adjusted for age, gender, residence, province, and waist circumference (P*-values) or hip circumference (P**-values) or waist-to-hip ratio (P***-values). Bold values indicate a statistically significant after adjustment for multiple testing (P-values < 0.025). (DOCX 19 kb) [file 12944_2016_349_MOESM2_ESM.docx]

**Table S2**. Multivariate analysis of association for dyslipidemia, adjusted for age, gender, residence, province and obesity-related traits

| Lipid disorder | *APOE genotype* | OR^*^ (95%CI) | *P^*^*-value | OR^**^ (95%CI) | *P^**^*-value | OR^***^ (95%CI) | *P^***^*-value |
| --- | --- | --- | --- | --- | --- | --- | --- |
| *Hypoalphalipoproteinemia*  *(N=33/600)* | ε3/ε3 | 1 |  | 1 |  | 1 |  |
|  | ε2 carrier | 1.27 (0.44-3.65) | 0.653 | 1.38 (0.48-3.95) | 0.550 | 1.36 (0.48-3.89) | 0.562 |
|  | ε4 carrier | 2.90 (1.17-7.23) | **0.022** | 2.81 (1.13-6.97) | 0.026 | 3.21 (1.29-8.00) | **0.012** |
| *Hyperbetalipoproteinemia*  *(N=35/600)* | ε3/ε3 | 1 |  | 1 |  | 1 |  |
|  | ε2 carrier | 0.76 (0.21-2.84) | 0.688 | 0.90 (0.24-3.33) | 0.871 | 0.85 (0.23-3.21) | 0.815 |
|  | ε4 carrier | 1.85 (0.70-4.87) | 0.212 | 2.04 (0.77-5.40) | 0.151 | 2.21 (0.84-7.79) | 0.107 |
| *Hypertriglycemia*  *(N=197/600)* | ε3/ε3 | 1 |  | 1 |  | 1 |  |
|  | ε2 carrier | 1.12 (0.69-1.83) | 0.650 | 1.18 (0.72-1.93) | 0.514 | 1.13 (0.70-1.84) | 0.614 |
|  | ε4 carrier | 1.01 (0.57-1.77) | 0.975 | 0.96 (0.54-1.69) | 0.882 | 1.03 (0.59-1.80) | 0.907 |
| *Hypercholesterolemia*  *(N=42/600)* | ε3/ε3 | 1 |  | 1 |  | 1 |  |
|  | ε2 carrier | 0.62 (0.18-2.13) | 0.446 | 0.67 (0.19-2.30) | 0.521 | 0.67 (0.20-2.31) | 0.527 |
|  | ε4 carrier | 1.71 (0.70-4.21) | 0.242 | 1.76 (0.71-4.36) | 0.225 | 1.80 (0.73-4.43) | 0.199 |
|  | T/T |  |  |  |  |  |  |
| Dyslipidemia  *(N=249/600)* | ε3/ε3 | 1 |  | 1 |  | 1 |  |
|  | ε2 carrier | 1.09 (0.69-1.72) | 0.716 | 1.14 (0.72-1.81) | 0.568 | 1.10 (0.70-1.73) | 0.680 |
|  | ε4 carrier | 1.25 (0.77-2.05) | 0.363 | 1.22 (0.74-1.99) | 0.437 | 1.28 (0.79-2.06) | 0.323 |
| *P*-values obtained by multivariate logistic regression and adjusted for age, gender, residence, province, and waist circumference (*P**-values) or hip circumference (*P***-values) or waist-to-hip ratio (*P****-values).  Bold values indicate a statistically significant after adjustment for multiple testing (*P*-values < 0.025). | | | | | | | |
